# IFNγ-primed periodontal ligament cells regulate T-cell responses via IFNγ-inducible mediators and ICAM-1-mediated direct cell contact

**DOI:** 10.1098/rsos.220056

**Published:** 2022-07-27

**Authors:** Weerachai Singhatanadgit, Setthawut Kitpakornsanti, Montree Toso, Prasit Pavasant

**Affiliations:** ^1^ Oral and Maxillofacial Surgery Unit, Faculty of Dentistry, Thammasat University, Rangsit Campus, Pathumthani, Thailand; ^2^ Research Unit in Mineralized Tissue Reconstruction, Thammasat University, Rangsit Campus, Pathumthani, Thailand; ^3^ Stem Cell for Life Research Center, Greater Pharma Manufacturing Co. Ltd, Nakhon Pathom, Thailand; ^4^ Center of Excellence in Regenerative Dentistry, Faculty of Dentistry, Chulalongkorn University, Bangkok, Thailand; ^5^ Department of Anatomy, Faculty of Dentistry, Chulalongkorn University, Bangkok, Thailand

**Keywords:** interferon γ, periodontal ligament cells, T lymphocytes, intercellular adhesion molecule-1, transforming growth factor β1, immunosuppression

## Abstract

Periodontal ligament (PDL) cells help maintain tissue homeostasis by balancing PDL tissue inflammation and regeneration. However, the mechanisms by which interferon γ (IFNγ) modulate this process are not yet fully understood. The present study aimed to examine the effect of primed and non-primed PDL cells with IFNγ on the viability and differentiation of T lymphocytes and its functional consequences. The results showed that IFNγ-primed PDL cells possessed enhanced immunosuppression by suppressing T-lymphocyte viability and directing T-lymphocyte differentiation towards a higher T helper (Th) Th2/Th1 ratio. Suppression of T-cell viability was mainly mediated by IFNγ-inducible secreted mediators, which was prevented in the presence of direct cell contact, probably by intercellular adhesion molecule-1 (ICAM-1)-induced PI3 K-mediated transforming growth factor β1 expression in PDL cells. By contrast, ICAM-1 activation augmented IFNγ-induced IFNγ and interleukin-6 expression in PDL cells, which in turn modulated T-cell differentiation. The resulting interaction between these two cell types activated macrophage and suppressed osteoclast differentiation. In conclusion, the results have shown, for the first time to our knowledge, that primed and non-primed PDL cells with IFNγ differentially control T-cell responses via IFNγ-inducible mediators and ICAM-1-mediated direct cell contact, suggesting the role of PDL cells in shifting an inflammatory phase towards a regenerative phase.

## 1. Introduction

When the periodontal ligament (PDL) is exposed to injuries such as microflora or mechanical forces, PDL cells help retain tissue homeostasis by balancing between tissue destruction and tissue regeneration during injury-induced tissue inflammation [1]. Cellular changes initiated by inflammation are associated with several inflammatory mediators and growth factors expressed and secreted by PDL cells. The biological characteristics of PDL cells are influenced by extracellular stimuli, such as inflammation [2,3], and this inflammatory process is a prerequisite for the adaptive response of PDL to stimuli [4]. Local synthesis and release of various mediators, including cytokines and growth factors, induce many cellular responses by various cell types, including PDL cells and inflammatory cells. This provides a favourable microenvironment, where cytokines and other associated molecules interact to allow cells to communicate with one another with and without direct cell contact, to maintain tissue homeostasis [4]. Clinical studies also suggested that high levels of inflammatory mediators and adhesion molecules were localized in inflamed periodontal tissues [5].

Interleukins (IL)-4, IL-6, interferon γ (IFNγ) and transforming growth factor β1 (TGFβ1) are among the cytokines found in an inflammatory PDL tissue. These mediators activate intracellular cascades and transcription factors that in turn attract more inflammatory cells and influence the healing of periodontal tissues [6]. This is also accompanied by an adaptive immune response, including differentiation of CD4^+^ T lymphocytes to T cell subsets, including T helper (Th) cells types 1 and 2 [7]. Th1 and Th2 lymphocytes produce various cytokines known to influence tissue remodelling, such as IFNγ, IL-4, IL-6 and TGFβ1, and thus play an important role in periodontal tissue homeostasis [8–11]. Non-immune cells, such as PDL cells, express a wide range of immunoregulatory molecules, and in response to inflammatory cytokines, such as IFNγ, PDL cells elicit their enhanced immunosuppressive role to properly control highly active immune cells involved in PDL homeostasis [12]. However, precise mechanisms remain unclear.

Although the immunomodulatory effect of mesenchymal stem cells (MSCs) is generally mediated via soluble factors, this could also be mediated through direct cell-to-cell contact [13]. In addition to its role as an adhesion molecule, intercellular adhesion molecule 1 (ICAM-1) mediates sensing of the inflammatory microenvironment via direct physical interaction with inflammatory cells, subsequently inducing intracellular signalling and modulating ICAM-1-dependent immunomodulatory capacities of MSCs [14]. ICAM-1 is an immunoglobulin (Ig)-like cell adhesion molecule expressed by a variety of cell types, including leukocytes and endothelial cells. Its key ligands, including membrane-bound integrin receptors, lymphocyte function-associated antigen-1 (LFA-1), are expressed mainly on the surface of leukocytes. It has been shown that upon binding to its cognate ligand, ICAM-1 initiates several intracellular signalling pathways, such as mitogen-activated protein kinases (MAPKs) and phosphoinositide 3-kinases (PI3 K), to control the expression of several genes associated with inflammation [15,16]. Furthermore, under an inflamed environment, it has been shown that increased expression of ICAM-1 by inflammatory cytokines, including IFNγ, enhances the immunosuppressive capacity of MSCs [17]. This suggests the involvement of ICAM-1, and further emphasizes the importance of IFN-γ, in the immunomodulatory role of MSCs.

MSCs’ immunosuppressive ability is induced in inflammatory circumstances [18]. MSCs that have been prestimulated with the proinflammatory cytokines IFNγ, tumour necrosis factor α (TNF-α) and IL-6 are more effective at inhibiting lymphocyte proliferation [19]. Furthermore, pretreatment with IFNγ improves the immunomodulatory action of MSCs by increasing cell–cell contact and the release of immunosuppressive soluble molecules [20,21]. Under inflammatory conditions, PDL cells, which show stem cell-like properties, may possess significant immunomodulatory roles. However, little is known about the precise mechanism of PDL cells-mediated T-cell responses, which can play an important part in PDL tissue homeostasis under the inflammatory microenvironment. The immunomodulatory function of PDL cells may help inhibit prolonged inflammation and immune responses and suppress osteoclast-mediated bone destruction.

It is thus possible that in response to injury-induced inflammation, PDL cells may possess a potent immunosuppressive property, but the precise mechanisms by which IFNγ modulates PDL cell-mediated tissue homeostasis are not yet fully understood. It is, however, hypothesized that IFNγ may regulate PDL cell-mediated immunosuppression and subsequent biological functions. The objective of the study, therefore, aimed to examine the effect of IFNγ-primed PDL cells on the viability and differentiation of T lymphocytes and its functional consequences on macrophage activation and osteoclast formation *in vitro*. The IFNγ-induced immunomodulatory molecules controlling these biological events were also investigated.

## 2. Material and methods

### 2.1. Isolation and culture of primary human T lymphocytes

Primary human T lymphocytes were isolated from buffy coats of fully anonymized healthy donors who provided blood donations to the Thai Red Cross Society, Bangkok, Thailand. The use of buffy coats was approved by the Ethics Review Sub-Committee for Research Involving Human Research Subjects and the Institutional Biosafety Committee Thammasat University. Permission for the use of buffy coats was obtained from the Thai Red Cross Society. The donors who have no clinical history of immunological abnormalities and infectious diseases were recruited. The buffy coats were kept at 18°C throughout the transfer from the Thai Red Cross Society to the laboratory at Thammasat University, where the samples were processed on the day of arrival. For T-lymphocyte isolation, the buffy coat was diluted 1 : 1 with phosphate-buffered saline (PBS), layered on Ficoll-Paque PLUS (GE Healthcare Life Sciences) and centrifuged at 800*g* for 40 min at 18°C. The peripheral blood mononuclear cell (PBMC) interface layer was collected, washed three times with PBS by centrifugation at 800*g* for 10 min at 18°C and placed into 75 cm^2^ culture flasks containing 10 ml of Dulbecco's modified eagle medium (Gibco Life Technologies Ltd, Paisley, UK) containing 10% fetal calf serum (FCS) supplemented with 200 U ml^−1^ penicillin, 200 µg ml^−1^ streptomycin, 2 mM l-glutamine (all from Gibco) (standard culture medium). After 2 h at 37°C to allow the monocytes to adhere, the non-adherent cells were collected and activated with 5 µg ml^−1^ concanavalin A (ConA) for 72 h to obtain activated T-lymphocyte cultures which were used in all subsequent experiments. The purity of the T cells used here was determined to be approximately 90%, and their activation was demonstrated by increases in CD69 expression and cell number (electronic supplementary material, table S1).

### 2.2. Isolation and culture of primary human periodontal ligament cells

The present study collected periodontally healthy teeth from patients undergoing routine extraction at the Oral Surgery Clinic, Dental Unit, Thammasat University Hospital. The participants were asked to sign informed consent to the use of these tissues following the protocol approved by the Ethics Review Sub-Committee for Research Involving Human Research Subjects and the Institutional Biosafety Committee Thammasat University. The PDL tissue was explanted to obtain PDL cells, and the cells were cultured in the standard culture medium at 37°C in a humidified atmosphere of 5% CO_2_ in air. PDL cells from three donors were successfully isolated, and cells between passages 3–6 were used.

### 2.3. Co-cultures of periodontal ligament cells and T cells

PDL cells were seeded onto well plates with a density of 5000 cells cm^−2^. The cells were treated for 48 h with IFNγ at indicated concentrations (0–100 ng ml^−1^) in a standard culture medium. Activated T cells were resuspended in a standard culture medium and added at indicated ratios of PDL cells: T cells ranging between 1 : 5 and 1 : 40. In some experiments, the two types of cells were separated to prevent direct physical contact by a semi-permeable porous membrane (0.4 µm) using a cell culture insert (Nunc).

### 2.4. Treatment of cells

Priming of PDL cells with IFNγ was performed by seeding PDL cells onto well plates with the density of 5000 cells cm^−2^ and cultured in standard medium for 24 h, and cells were treated for 48 h with IFNγ at indicated concentrations (0–100 ng ml^−1^) in standard culture medium. Cells were washed three times with a standard culture medium to eliminate exogenously added IFNγ before further experiments.

To block the activity of biological molecules of interest, the following neutralizing antibodies were used to pretreat cell cultures for 30 min before further treatments/assays: monoclonal antibodies (mAb) specific to ICAM-1 (5 µg ml^−1^, mAb IgG1 clone no. BBIG-I1 (11C81), R&D systems), LFA-1 (10 µg ml^−1^ mAb IgG1 clone TS1/22, Thermo scientific), IL-4 (1 µg ml^−1^, IgG2B clone no. 34019, R&D systems) and IL-6 (0.5 µg ml^−1^, IgG1 clone no. 6708, R&D systems) and a polyclonal antibody specific to TGFβ1 (0.2 µg ml^−1^, polyclonal chicken IgY, catalogue no. AB-101-NA, R&D systems). Corresponding isotype controls were also used (R&D Systems).

To activate ICAM-1 intracellular signalling in IFNγ-primed PDL cells, PDL cells were primed with IFNγ as detailed above and incubated with mouse anti-human ICAM-1 mAb (5 µg ml^−1^, mAb IgG1 clone no. BBIG-I1 (11C81), R&D systems), for 20 min at room temperature, followed by goat anti-mouse polyclonal IgG (GAM, 10 µg ml^−1^, Thermo Scientific) for cross-linking at 37°C for the duration indicated in each experiment.

In some experiments, PDL cells were pretreated with SB203580 (10 µM), SP60012 (10 µM), U0126 (1 µM) and wortmannin (0.5 µM) (all from Sigma), which are potent inhibitors specific to p38 MAPK, Jun N-terminal kinases (JNK) 1/2/3, extracellular regulated kinases (ERK) 1/2 and PI3 K, respectively, for 30 min before cross-linking of ICAM-1 in PDL cells to explore the involvement of these signalling pathway(s) in ICAM-1-mediated immunoregulatory effect of IFNγ-primed PDL cells.

### 2.5. Cell viability assay

The effect of IFNγ on the viability of PDL cells was determined using the alamarBlue Assay (Invitrogen) according to the manufacturer's protocol. The samples were spectrophotometrically measured using a spectrophotometer at 560 nm and 600 nm wavelengths, and the cell viability was calculated following the manufacturer's instructions.

The viability of T cells in co-cultures was measured by using a trypan blue exclusion assay. Single-cell suspension of non-adherent T cells and T cells adhered on PDL cells, which were harvested by using the dissociation reagent TrypLE Express (Gibco) for 5 min at 37°C with gentle pipetting, were subjected to the trypan blue exclusion test, and the total number of viable T cells in each sample was calculated. Following the dissociation procedure used, detached PDL cells were distinguishable from T cells by their shape and size under light microscopy

For RAW 246.7 cell viability assay, 3-(4,5-dimethylthiazol-2-yl)-2,5-diphenyltetrazolium bromide (MTT) assay was used. After being activated and treated with various conditioned media, the samples were incubated with MTT solution (0.2%) at 37°C for 4 h, followed by adding dimethyl sulfoxide and glycine buffer. The end product solution was analysed by measuring absorbance at 490 nm (A_490_), which corresponds to the cell viability.

### 2.6. Scanning electron microscopy

Monolayers of PDL cells were obtained by seeding the cells on sterile plastic coverslips (Thermanox, NUNC; Naperville, IL, USA) at a density of 1 × 10^4^ cells cm^−2^ and incubating for 24 h. After removing the culture medium, suspensions of T cells (1 × 10^6^ cells ml^−1^) were seeded onto the PDL cells, and the co-cultures were incubated in a standard medium for 72 h. After washing extensively with PBS to remove non-adherent T cells, the samples were then fixed overnight in 3% glutaraldehyde in 0.14 M sodium cacodylate buffer (pH 7.3), dehydrated in a graded sequence of alcohols (50%, 70%, 90% and two changes of 100%), rinsed with PBS for 5 min, and deposited in a desiccator. After 24 h, the dehydrated samples were mounted onto stubs and then sputter-coated with gold/palladium using a Polaron E5100 coating device. Cell morphology and direct cell contact were observed using a JEOL JSM 5410LV scanning electron microscope (JEOL UK, Welwyn Garden City, UK).

### 2.7. RNA extraction and quantitative real-time reverse transcription-polymerase chain reaction

Total RNA was isolated using the RNeasy Mini Kit (Qiagen, CA, USA), and first-strand complementary DNA (cDNA) was synthesized from 1 µg RNA using a cDNA Synthesis Kit (Thermo Scientific). The first-strand cDNA was subjected to quantitative polymerase chain reaction (qPCR) using SYBR Green I dye, as suggested by the manufacturer (Roche Diagnostics GmbH, Mannheim, Germany) performed in an iQ5 iCycler (Bio­RAd, Bradford, UK), with specific primers (table 1) [22,23]. Glyceraldehyde 3-phosphate dehydrogenase (GAPDH) and 18 s rRNA were used as endogenous controls. The amplification conditions consisted of 40 cycles at 95°C for 15 s, followed by 60°C for 30 s and subsequently 72°C for 30 s. The specificity of the PCR products was verified by melting curve analysis. PCR reactions were performed in six replicates, and each of the gene signals was normalized to either GAPDH or 18 s rRNA signal in the same reaction. Three biological replicates were used to assess the biological variability of the gene expression results. All biological replicates provided similar gene expression patterns.

**Table 1.** Primer sequences used in the study.

genes	forward sequences	reverse sequences
human GAPDH	5′-CTGGGCTACACTGAGCACC-3′	5′-AAGTGGTCGTTGAGGGCAATG-3′
human 18 s rRNA	5′-TGCCTTCCTGGATGTGGTAG-3′	5′-CGTCTGCCCTATCAACTTTCG-3′
human IFNγR1	5′-GGCAGCATCGCTTTAAACTC-3′	5′-AGGTGGGGGCTTTTATTACG-3′
human IFNγR2	5′-AGTCCAGGCACAACTGCTTT-3′	5′-AATGTTCCCACGGAGATCAG-3′
human IFNγ	5′-CTAGGCAGCCAACCTAAGCA-3′	5′-CAGGGTCACCTGACACATTC-3′
human STAT-1	5′-CAATGGTGTGGCAAAGAGTG-3′	5′-GGGCATTCTGGGTAAGTTCA-3′
human TBX21	5′-GTCCAACAATGTGACCCAGAT-3′	5′-ACCTCAACGATATGCAGCCG-3′
human GATA3	5′-GCCCCTCATTAAGCCCAAG-3′	5′-TTGTGGTGGTCTGACAGTTCG-3′
human IL-4	5′-CTCATTTTCCCTCGGTTTC-3′	5′-GAAGCAGTTGGGAGGTGAG-3′
human IL-6	5′-TTCAATGAGGAGACTTGCC-3′	5′-TGACCAGAAGAAGGAATGA-3′
human TGFβ1	5′-CTAATGGTGGAAACCCACAACG-3′	5′-TATCGCCAGGAATTGTTGCTG-3′
human ICAM-1	5′-AGACATAGCCCCACCATGAG-3′	5′-TCAAGGGTTGGGGTCAGTAG-3′
human TRAP	5′-GACTGTGCAGATCCTGGGTG-3′	5′-GGTCAGAGAATACGTCCTCAAAG-3′
human CTSK	5′-ACTCAAAGTACCCCTGTCTCAT-3′	5′-ACTCAAAGTACCCCTGTCTCAT-3′
mouse GAPDH	5′-AGGTCGGTGTGAACGGATTTG-3′	5′- TGTAGACCATGTAGTTGAGGTCA-3′
mouse IL-1β	5′-GCAACTGTTCCTGAACTCAACT-3′	5′-ATCTTTTGGGGTCCGTCAACT-3′
mouse TNFα	5′-CCCTCACACTCAGATCATCTTCT-3′	5′-GCTACGACGTGGGCTACAG-3′

### 2.8. Separation of T cells from co-cultures using CD3 magnetic microbeads

T cells were separated from T cell-PDL cell co-cultures using human CD3 magnetic microbeads (Miltenyi Biotech, CA, USA) by following the manufacturer's instructions. In brief, a single-cell suspension (80 µl) of approximately 10^7^ cells in PBS containing 0.5% bovine serum albumin (BSA) and 2 mM ethylene diamine tetraacetic acid (EDTA) (buffer solution) was incubated with a CD3 microbead aliquot (20 µl) for 15 min at 4°C. The sample was then washed by adding 2 ml of buffer solution and centrifuged at 300 g for 10 min. The sample was resuspended in 500 µl of buffer solution, and the magnetically labelled Tcell fraction was collected for further analyses.

### 2.9. Flow cytometry

For cell cycle analysis, cells were collected following co-culture for 24 h, and the cell pellets were resuspended in ice-cold 70% ethanol for 30 min. Cells were then rinsed twice in PBS and resuspended in 20 µg ml^−1^ propidium iodide in PBS with 50 µg ml^−1^ RNase A (both from Sigma) at 4°C for 30 min and subjected to flow cytometry (FCM). The distribution of T cells in three major phases of the cycle (G0/G1 versus S versus G2/M) was analysed. For cell proliferation assay using 5-(and 6)-carboxyfluorescein diacetate succinimidyl ester (CSFE) labelling and FCM, 10^7^ T cells were labelled with 1 µM CSFE (eBioscience) for 10 min at room temperature in the dark, and CFSE-labelled and unlabelled cells were activated with 5 µg ml^−1^ ConA for 72 h. The cells were further co-cultured with PDL cells for another 72 h in the dark before subjecting to FCM. The proliferation index (PI) was analysed using the ModFit LT 5.0 program (Verity Software House, ME, USA), which automatically calculated the PI of T cells as the sum of cells in all generations divided by the number of original parent cells. Apoptosis of T cells was measured, following 24 h in co-culture, by staining the cells with annexin V-fluorescein-5-isothiocyanate (V-FITC) and propidium iodide (Miltenyi Biotech) by following the manufacturer's instruction, and the stained T cells were analysed by FCM. The annexin V^+^PI^−^ and annexin V^+^PI^+^ T cells were considered to be apoptotic cells. Analysis of cell cycle, PI and apoptosis, carried out on T-cell gating, is useful for determining anti-proliferative effects of PDL cells (both non-primed and IFNγ-primed) on T cells.

FCM was also used to examine the expression of CD3, CD4, CD8, IFNγ and IL-4 in T cells and the expression of TGFβ1 and ICAM-1 in PDL cells. For the detection of intracellular IFNγ, IL-4 and TGFβ1 antigens, 1 µM of monensin (Sigma) was added to the cultures for the last 6 h of incubation. The T-cell suspensions were stained with anti-CD3-ECD, -CD4-PC5 and -CD8-PE antibodies (Beckman Counter) following the manufacturer's instruction. The samples were then fixed with 1% paraformaldehyde (and permeabilized with 0.1% saponin for intracellular antigens). They will be washed with PBS containing 1% FCS (PBS-FCS) after each step described below. Following centrifugation, cells were blocked with 1% human serum in PBS for 30 min at room temperature and stained with appropriate fluorescence-conjugated antibodies specific to each of the above antigens mentioned, i.e. anti-IFNγ-FITC and -IL-4-PE-Vio 770 antibodies (Miltenyi Biotech), diluted in PBS (pH 7.2) containing 0.5% BSA and 2 mM EDTA. Corresponding fluorescence-conjugated isotype control antibodies (BD Pharmingen) were used as the negative control. For PDL cells, detection of total TGFβ1 and cell surface ICAM-1 antigens were performed in permeabilized and non-permeabilized samples, respectively, following the staining protocol described above. Samples were stained with anti-TGFβ1 and anti-ICAM-1 primary antibodies, followed by the corresponding fluorescence-conjugated secondary antibodies (all from R&D), according to the manufacturer's instructions.

For data acquisition and analysis, all FCM experiments were performed using the CytoFLEX Flow Cytometer and the CytExpert software (both from Beckman Coulter, CA, USA). For multicolour FCM, fluorescence compensation setting was first determined and kept constant in all experiments.

### 2.10. Western blot analysis

Total protein was extracted from cells using a radio-immunoprecipitation assay (RIPA) cell lysis buffer containing protease and phosphatase inhibitor cocktails (Thermo Fisher Scientific). The total protein amount was quantified in RIPA extracts using the bicinchoninic acid kit. Equivalent quantities of RIPA-solubilized proteins were resolved by 7–14% sodium dodecyl sulfate/polyacrylamide gels, and the separated proteins were transferred to nitrocellulose membranes (Merck Millipore, MA, USA). Membranes were blocked with 5% non-fat milk in tris-buffered saline with 0.1% Tween 20 (TBST) and probed with the following primary antibodies: anti-p38 MAPK, anti-JNK, anti-ERK, anti-PI3 K, anti-phospho-p38 MAPK Thr180/Tyr182 (p-p38), anti-phospho-SAPK/JNK Thr183/Tyr185 (p-JNK), anti-phospho-ERK1/2 MAPK Thr202/Tyr204 (p-ERK), anti-phospho-PI3 K Tyr458/Tyr199 (p-PI3 K) (all from Cell Signalling Technology) and anti-α-tubulin (Santa Cruz) diluted 1 : 1000 in 5% BSA in TBST overnight at 4°C. Anti-rabbit IgG-horseradish peroxidase (Santa Cruz) diluted 1 : 5000 in 5% BSA in TBST was used to visualize primary antibody-probed blots. Autoradiography with increased chemoluminescence was used to identify proteins (Merck Millipore). The intensities of protein bands were evaluated using the ImageJ software (National Institutes of Health, MD, USA) after the films were scanned and stored as digital pictures.

### 2.11. Preparation of conditioned media

Following treatment of PDL cell monoculture, cells at 70–80% confluent were washed with PBS and cultured in a standard culture medium. The medium was collected after 48 h, centrifuged for 5 min at 400*g* (4°C) and then for another 3 min at 2000*g* (4°C) to remove cells, filtered, and stored at −80°C within one month until use. The conditioned medium derived from a co-culture was also collected after 48 h and prepared as described above.

### 2.12. Cytokine depletion

Cytokines were depleted from the conditioned medium using anti-human polyclonal antibodies against IFNγ, IL-4 and TGFβ1 (all from R&D). Briefly, Protein G sepharose (GE Healthcare Ltd, Little Chalfont, UK) was pre-coated with each antibody at a concentration of 1 µg ml^−1^ and gently mixed with a conditioned medium overnight at 4°C, using a rotator. The samples were then centrifuged at 1700*g* for 5 min at 4°C to remove the beads. Enzyme-linked immunosorbent assay (ELISA) was used to confirm depletion (60–80% reduction).

### 2.13. Enzyme-linked immunosorbent assay

The levels of IFNγ, IL-4, IL-6 and TGFβ1 were investigated using ELISA. The concentration of these factors was measured using the Human Quantikine ELISA kits (R&D Systems) according to the manufacturer's instructions.

### 2.14. Analysis of macrophage activation and phagocytosis

RAW 246.7 monocyte/macrophage-like cells were activated with lipopolysaccharide (LPS; 100 ng ml^−1^, Sigma) with and without diluted (1 : 1) conditioned media collected from 24 h cultures of T cells alone (T CM), PDL cells and T cells (PDL-T CM) and IFNγ-primed PDL cells and T cells (IFNγ-PDL-T CM). After 24 h in culture, cell morphology was observed under a light microscope (Nikon Eclipse TS100), and photomicrographs of osteoclast appearances were taken using a Nikon Digital sight DS-L2. The number of viable cells was determined by MTT assay, and the expression of IL-1β and TNFα genes was determined by qPCR, as described above. For a phagocytosis assay, LPS-activated RAW 246.7 cells were co-cultured with heat-killed CFSE-stained *Streptococcus sanguinis*, at a concentration of 10 bacteria per one RAW 246.7 cell, for 60 min to allow phagocytosis. The levels of CFSE^+^ and CFSE^−^ RAW 246.7 cells were determined by FCM. Confocal fluorescence of paraformaldehyde-fixed culture stained with 0.1 nM rhodamine phalloidin (Thermo Fisher Scientific, Waltham, MA, USA) and 1 µM DAPI (Sigma) was also carried out to confirm cellular localization of CSFE-stained bacteria in RAW 246.7 cells. CSFE-stained bacteria were stained green, while nuclei and intracellular actin filaments were stained blue and red, respectively.

### 2.15. Osteoclast formation assay

PBMCs, isolated as described above, were used in this assay. Cells were induced with osteoclastic medium (OCM), consisting of 25 µg ml^−1^ macrophage colony-stimulating factor (MCSF) and 25 µg ml^−1^ receptor activator of NF-κB ligand (RANKL), in the presence of 1 : 1 diluted conditioned media collected from 24 h cultures of T CM, PDL-T CM and IFNγ-PDL-T CM, for 14 days. The presence of tartrate-resistant acid phosphatase (TRAP^+^) multinucleated cells was observed under a light microscope (Nikon Eclipse TS100), and photomicrographs of osteoclast appearances were taken using a Nikon Digital sight DS-L2. The number of osteoclasts (TRAP^+^ cells with more than two nuclei) per area (mm^3^) was also counted manually. The expression of osteoclast differentiation TRAP and cathepsin K (CTSK) genes was determined by qPCR, as described above.

### 2.16. Statistical analysis

The data are presented as the mean ± s.d. from PDL cells obtained from three donors, with the experiments being performed at least three times independently for each line of the PDL cells. Statistical differences were analysed by one-way ANOVA, followed by the *post hoc* Bonferroni test, with *p* < 0.05 considered statistically significant. The one-way ANOVA and Bonferroni test in the SPSS software were used for the analyses.

## 3. Results

### 3.1. Periodontal ligament cells were responsive to IFNγ and physically interacted with T cells *in vitro*

**Figure 1.**
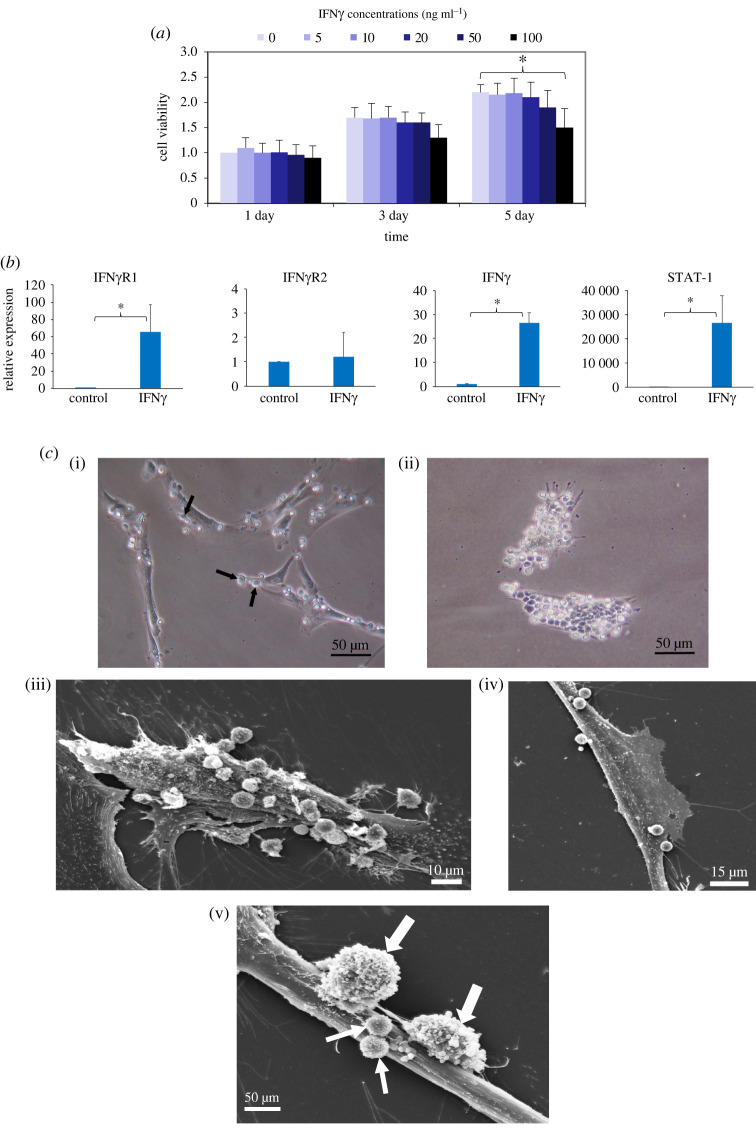
Expression of IFNγ signalling molecules in PDL cells, its effect on PDL cell viability, and the presence of physical contact between PDL cells and T cells *in vitro*. (*a*) Effect of IFNγ on the viability and proliferation of PDL cells. Cells were treated with IFNγ at various concentrations and time points. The samples were then subjected to the alamar blue cell viability assay. The results are presented as the mean ± s.d. of the viability level from three biological replicates (three donors), defined as 1.0 in the control cells treated with IFNγ 0 ng ml^−1^ for 1 day. (*b*) PDL cells were treated with 50 ng ml^−1^ IFNγ for 48 h and were subjected to RNA extraction and real-time qPCR. The results are presented as the mean ± s.d. of the level of each transcript relative to that of 18S rRNA from three biological replicates (three donors), defined as 1.0 in control untreated cells. (*c*) Representative light microscopy (i–ii) and scanning electron microscopy (iii–v) micrographs demonstrating direct cell-to-cell interaction between PDL cells and activated T lymphocytes (black arrows) after co-culture at PDL cell : T-cell ratio of 1 : 10 for 24 h. Note that the number of T lymphocytes adhering on a PDL cell varied from less than 10 to more than 15 cells ((i) versus (ii) and (iii) versus (iv)) and that within a PDL cell, both highly activated T lymphocytes (large white arrows) and possibly less activated T lymphocytes (small white arrows) were observed (v). *p < 0.05.

First, the effect of IFNγ on the viability and proliferation of PDL cells was examined, and the results are shown in figure 1*a*. Among all the concentrations and durations of IFNγ treatment, IFNγ at 100 ng ml^−1^ appeared to significantly reduce the number of viable PDL cells on day 5 compared with the untreated cells on day 5 by approximately 25% (figure 1*a*). The viable cells in the samples treated with this concentration only slightly increased from days 1 to 3, compared with the cells treated with IFNγ at lower concentrations, suggesting anti-proliferative, but not cytotoxic, activity of IFNγ at 100 ng ml^−1^ in PDL cells after 5 days in culture.

We further examined the responsiveness of PDL cells to an optimal concentration at 50 ng ml^−1^ of IFNγ. The results show that the transcripts of key molecules tested; IFNγR1, IFNγR2, IFNγ and STAT-1 were expressed in PDL cells (figure 1*b*). In addition, IFNγ treatment significantly and dramatically upregulated IFNγR1, but not IFNγR2, IFNγ and STAT-1 transcripts in PDL cells by approximately 60-, 24- and 25 000-fold, respectively, compared with the untreated cells (figure 1*b*). This suggests autocrine and paracrine actions of IFNγ on PDL cells.

The presence of direct contact of PDL cells and activated T lymphocytes was investigated in co-cultures by using light microscopy and scanning electron microscopy (SEM) (figure 1*c*). After 24 h in co-cultures, some T lymphocytes adhered to the PDL cell surface and were not dissociated by extensive washing. Approximately 5–25 T lymphocytes (e.g. black arrows in (i)) were estimated to be in contact with a PDL cell. The number of T lymphocytes adhering on a PDL cell varied from less than 10 to more than 15 cells, as shown by light microscopy and SEM (figure 1*c*(i) versus *c*(ii) and *c*(iii) versus *c*(iv)). A high magnification SEM micrograph in figure 1*c*(v) demonstrates the presence of two apparently activated T lymphocytes with characteristic extended cytoplasmic processes (large white arrows) very closely associated with the PDL cell and possibly less activated T lymphocytes (small white arrows). The majority of cells adhering on the PDL cells (96% of total cells adhering on PDL cells) were found to be CD3-positive, confirming T lymphocytes in origin (electronic supplementary material, table S2). No lymphocytes were observed in areas that did not contain adherent PDL cells. These provide evidence suggestive of direct contact between PDL cells and T lymphocytes.

Taken together, the results suggest that IFNγ at concentrations ranging from 5 to 50 ng ml^−1^ had little, if any, significant effect on PDL cell viability and proliferation after 5-day treatment and that PDL cells were responsive to IFNγ, possibly acting in autocrine and paracrine manners. Moreover, PDL cells interacted physically with activated T lymphocytes by allowing direct T-cell attachment ranging from a few to more than 15 T cells per a PDL cell.

### 3.2. The inhibitory role of IFNγ-primed periodontal ligament cells in T-cell viability depended on IFNγ-inducible mediators and periodontal ligament cell–T-cell interaction

**Figure 2.**
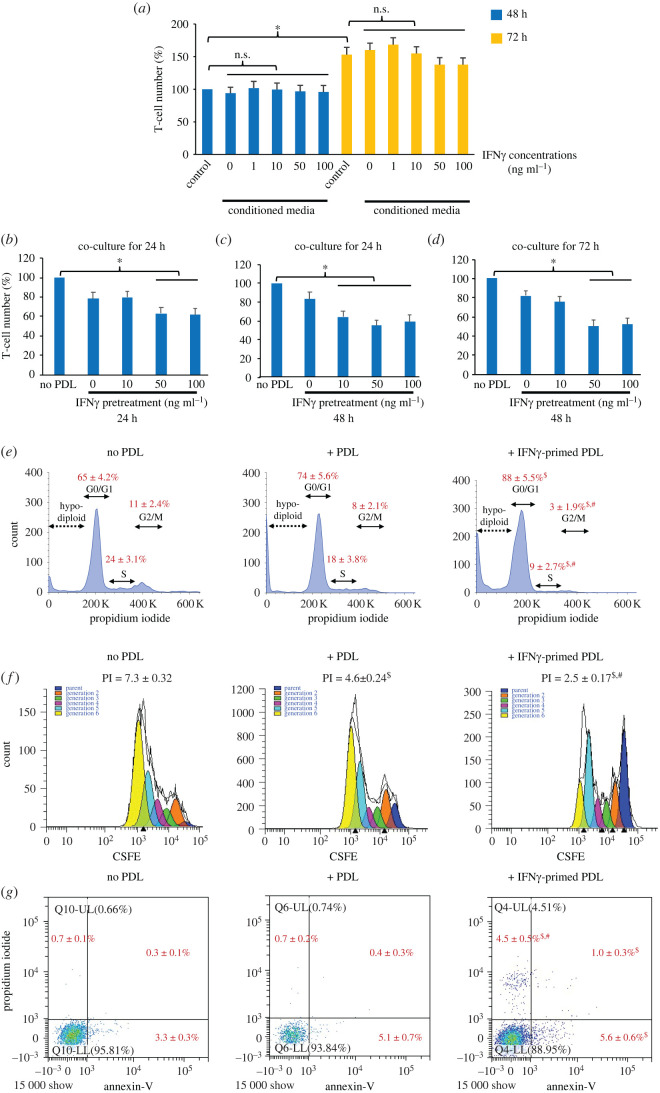
Effect of IFNγ-primed PDL cells on T-cell viability. (*a*) Effect of conditioned media derived from IFNγ-primed and non-primed PDL cells on T-cell viability. PDL cells were treated with IFNγ at various concentrations (0–100 ng ml^−1^) for 48 h, and they were cultured in a standard culture medium without IFNγ for another 48 h. The resulting PDL cell-conditioned media were collected and diluted 1 : 1 with a fresh standard culture medium, and the diluted condition media were used to treat T cells for 48 h and 72 h. The viability of T lymphocytes was determined by the trypan blue exclusion test. The standard culture medium was used as a control. The results are presented as the mean percentage ± s.d. of viable T cells from three biological replicates, defined as 100% in the 48 h control group. (*b–g*) Suppression of T-cell viability by IFNγ-primed PDL cells in co-cultures with T cells (in the presence of direct cell-to-cell contact). PDL cells were pretreated with IFNγ (0–100 ng ml^−1^) for 24–48 h, and they were co-cultured with T cells at a PDL : T cell ratio of 1 : 5 for indicated times (24 h or 72 h). The number of viable T cells was examined by using the trypan blue exclusion test (*b*–*d*). Cell cycle, proliferation index (PI) and apoptosis of T-cell monoculture, co-cultured with non-primed PDL cells and co-cultured with PDL cells primed with 50 ng ml^−1^ IFNγ for 48 h, were analysed using FCM (*e*–*g*, respectively). The results are presented as the mean ± s.d. of the values obtained from the experiments using PDL cells from three donors. *p < 0.05. NS, no significance. ^$^*p* < 0.05 versus no PDL control. ^#^*p* < 0.05 versus non-primed PDL group.

We tested whether the soluble mediators secreted from PDL cells pretreated with IFNγ at 1–100 ng ml^−1^ had any effect on the viability of activated T cells. The effect of conditioned media derived from IFNγ-primed and non-primed PDL cells on T-cell viability was therefore investigated. The results in figure 2*a* show that the viability of T cells was not affected by any of the PDL conditioned media for 48–72 h (figure 2*a*). It is important to note that the number of viable T cells increased by approximately 150% from 48 h to 72 h in culture, confirming the proliferative ability of the activated T cells used here.

The lack of suppressive effect of PDL cells and IFNγ-primed PDL cells on T-cell viability raises the possibility that an absence of certain mediators and PDL cell–T-cell interaction in the experiment using conditioned media could influence the immunosuppressive role of (IFNγ-primed) PDL cells. We, therefore, examined whether PDL cells and IFNγ-primed PDL cells possessed T-cell suppression potential in direct co-cultures of these two cell types. The results showed that after 24 h in co-cultures, 10–20% suppression of T-cell viability by PDL cells was observed, and IFNγ priming of PDL cells further suppressed T-cell viability significantly to 40–50% of the control, depending on the IFNγ concentration and duration used in priming the PDL cells (figure 2*b,c*). However, longer co-culture time (72 h) did not result in fewer T cells (figure 2*d*). Induction of cell cycle arrest, suppression of proliferation and stimulation of apoptosis of T cells caused by PDL cells and, even more pronounced, by IFNγ-primed PDL cells were evident (figure 2*e–g*, respectively). Although not statistically significant, a trend suggested that after co-cultured with PDL cells, the proportions of T cells in S and G2M phases were decreased, with the proportion in G0/1 being increased, compared with those of the T-cell culture alone (figure 2*e*). The results also showed that IFNγ priming further enhanced the suppressive role of PDL cells in the cell distribution in S and G2M phases significantly. Figure 2*f* shows that PDL cells and IFNγ-primed PDL cells significantly reduced T-cell proliferation by decreasing the PI from 7.3 in the control T cells alone to 4.6 in the co-cultures and that IFNγ priming significantly enhanced the inhibitory effect of PDL cells on the PI of T cells (4.6 versus 2.5). Although PDL cells only slightly caused early T-cell apoptosis, IFNγ priming significantly enhanced early T-cell apoptosis induced by PDL cells (figure 2*g*). In addition, only PDL cells that were previously primed with IFNγ were able to increase the late stage of apoptosis and necrosis of T cells (figure 2*g*).

The results indicate that only in direct co-cultures, PDL cells decreased the number of viable T cells and priming with IFNγ at 50–100 ng ml^−1^ for 48 h enhanced this suppressive effect, suggesting the involvement of IFNγ-inducible mediators and PDL cell–T-cell interaction in regulating T-cell number by PDL cells.

### 3.3. IFNγ-primed periodontal ligament cells regulated Th1/Th2 differentiation

**Figure 3.**
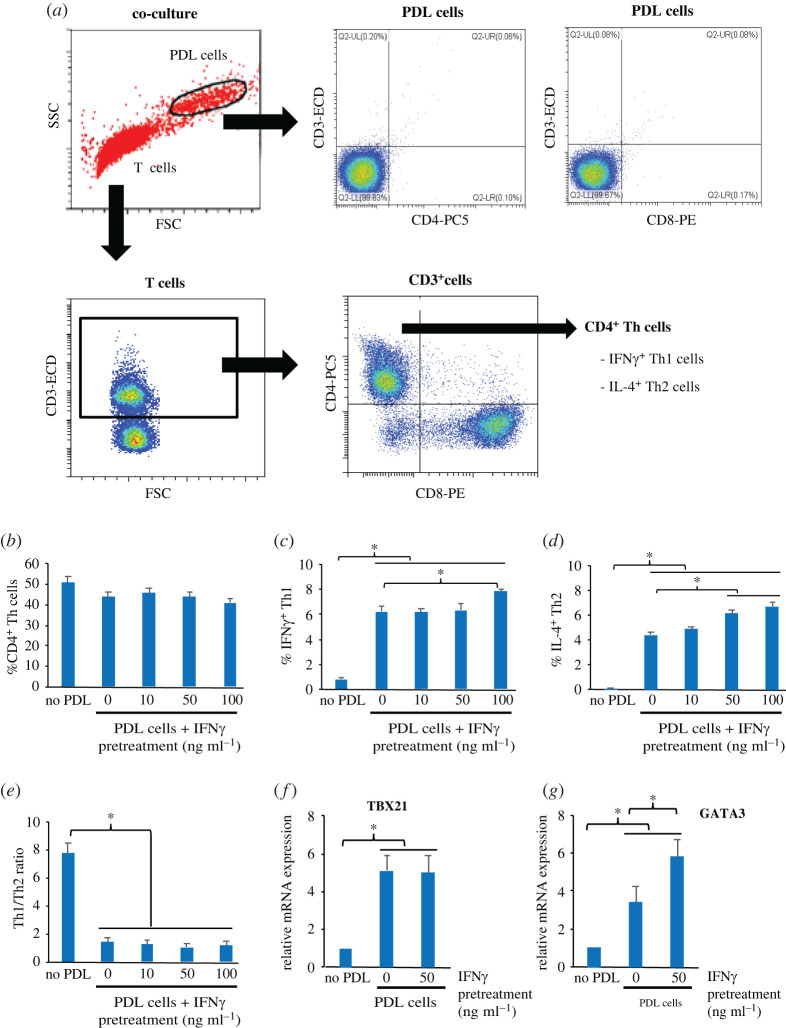
Effect of IFNγ-primed PDL cells on T-cell fates towards IFNγ^+^Th1 and IL-4 ^+^ Th2 lineages. (*a*) Flow cytometric gating strategy of a T-cell sample to identify Th cells (CD3 ^+^ CD4 ^+^ CD8^−^), Th1 cells (CD3 ^+^ CD4 ^+^ CD8^−^ IFNγ^+^ subpopulation) and Th2 cells (CD3 ^+^ CD4 ^+^ CD8^−^IL-4^+^ subpopulation). (*b–e*) Effect of IFNγ-primed PDL cells on Th1 and Th2 subpopulations. PDL cells were primed with 0–100 ng ml^−1^ IFNγ for 48 h and subsequently co-cultured with T cells (1 : 5) for 24 h, and the percentages of each T-cell subset were analysed by using immunostaining and flow cytometry. The percentages of Th, Th1 and Th2 cells and the Th1 : Th2 ratio are shown in (*b*–*e*), respectively. The results are presented as the mean percentage ± s.e. of the values obtained from three biological replicates. (*f–g*) Effect of IFNγ-primed PDL cells on the mRNA expression of TBX21 and GATA3 in T cells. PDL cells were primed with 0–100 ng ml^−1^ IFNγ for 48 h and then co-cultured with T cells (1 : 5) for 24 h. The samples were subjected to T-cell separation using CD3 bead assay, RNA extraction, and qPCR for the expression of TBX21 and GATA3 transcripts. The results are presented as the mean ± s.d. of the level of each transcript relative to that of 18S rRNA, defined as 1.0 in control untreated cells, obtained from three donors. *p < 0.05.

The effect of non-primed PDL and IFNγ-primed PDL cells on Th1 and Th2 differentiation in co-cultures was investigated using FCM. The gating strategy to identify these T-cell subpopulations is shown in figure 3*a*, and the CD3 ^+^ CD4 ^+^ CD8^−^ Th cells expressing IFNγ were defined as Th1 cells, while IL-4^+^ Th cells were defined as Th2 cells in our study. The results showed that following co-cultures for 24 h, both non-primed PDL cells and IFNγ -primed PDL cells had little effect on the level of CD4^+^ Th cells (figure 3*b*) but significantly increased the percentages of both Th1 cells and Th2 cells (figure 3*c,d*). However, only high doses of IFNγ at 100 ng ml^−1^ and 50–100 ng ml^−1^ further increased percentages of Th1 and Th2 cells, respectively, compared with the non-primed PDL cells. However, non-primed PDL cells and IFNγ-primed PDL cells dramatically reduced the proportion of Th1cells/Th2 cells by more than fourfold (figure 3*e*). The upregulation of Th1-specific transcription factor TBX21 and Th2-specific transcription factor GATA3 in T cells were observed in co-cultures (figure 3*f,g*), supporting increased populations of Th1 and Th2 cells induced by IFNγ-primed PDL cells. The results demonstrated that in direct co-cultures, IFNγ-primed PDL cells stimulated the differentiation of both Th1 and Th2 cells but to different extents, resulting in a higher Th2/Th1 ratio.

### 3.4. IFNγ upregulated the expression of immunoregulatory mediators IFNγ, IL-4, IL-6, TGFβ1 and ICAM-1 in periodontal ligament cells

**Figure 4.**
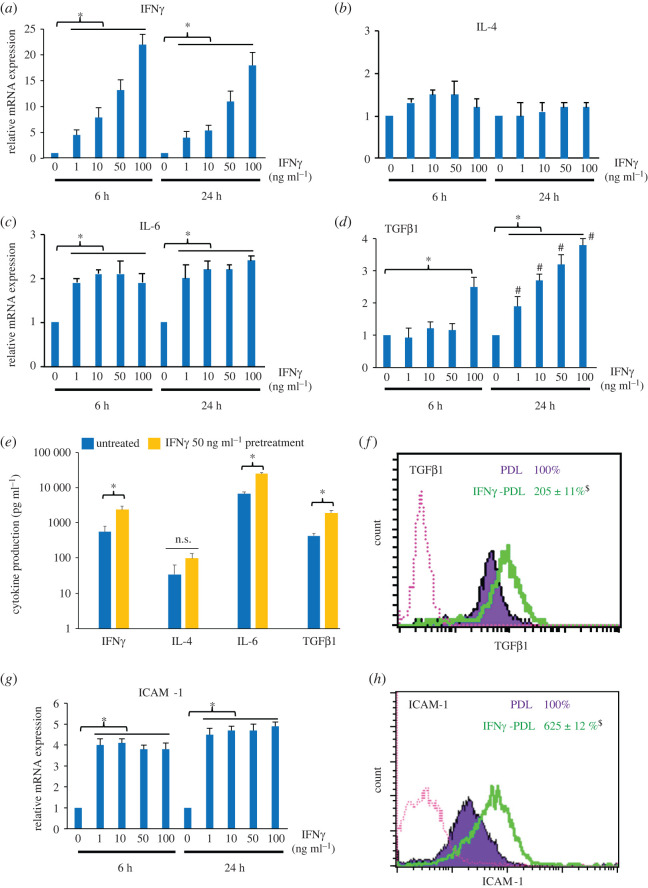
Effect of IFNγ on the expression of IFNγ, IL-4, IL-6, TGFβ1 and ICAM-1 in PDL cells. PDL cells were treated with IFNγ at various concentrations (0–100 ng ml^−1^). After 6 and 24 h, the cell samples were subjected to RNA extraction, and the mRNA expression of IFNγ, IL-4, IL-6, TGFβ1 and ICAM-1 was determined by qPCR (*a–d* and *g*). After 48 h of IFNγ treatment, cells were incubated with fresh culture medium for another 48 h when the concentration (in pg ml^−1^) of secreted protein products of IFNγ, IL-4, IL-6 and TGFβ1 present in conditioned media were measured by ELIZA (*e*). FCM was used to determine the expression of cellular latent TGFβ1 (*f*) and cell surface ICAM-1 (*h*). The qPCR (FCM) results are presented as the mean ± s.d. of the level of each transcript relative to that of 18S rRNA (of each cellular antigen), defined as 1.0 (100) in control untreated cells, from three donors. *p < 0.05. ^#^*p* < 0.05 versus 6 h group of at similar IFNγ concentration. ^$^*p* < 0.05 versus untreated control. n.s., no significance.

As the results suggest a possible involvement of IFNγ-inducible mediators and cell interaction in regulating the viability and differentiation of T cells by IFNγ-primed PDL cells, the effect of IFNγ on the expression of IFNγ, IL-4, IL-6, TGFβ1 and ICAM-1 in PDL cells was determined. These molecules are known IFNγ-inducible factors that play an important role in immunomodulation [24,25]. The qPCR results in figure 4 show that in response to IFNγ, the expression of immunomodulatory factors IFNγ, IL-6, TGFβ1, but not IL-4, messenger RNA (mRNA) transcripts were significantly upregulated in a dose–dependent manner in PDL cells from all three donors, but to different extents (figure 4*a–d*). The expression of TGFβ1 induced by IFNγ appeared to be time-dependent (figure 4*d*). The increased secreted levels of IFNγ, IL-6 and TGFβ1 proteins induced by IFNγ treatment (50 ng ml^−1^) were also confirmed by ELIZA (figure 4*e*), and the cellular level of the latent form of TGFβ1 was also induced, as observed by FCM (figure 4*f*). Moreover, the levels of ICAM-1mRNA and cell surface antigen were also stimulated by IFNγ independent of IFNγ concentration (figure 4*g,h*). The results indicated that IFNγ-primed PDL cells expressed elevated mRNA and protein expression of immunoregulatory mediators IFNγ, IL-4, IL-6, TGFβ1 and ICAM-1 compared with those in non-primed PDL cells.

### 3.5. Suppression of T-cell viability by IFNγ-primed PDL cells was prevented by ICAM-1-induced PI-3 K-mediated inhibition of TGFβ1 expression in periodontal ligament cells

**Figure 5.**
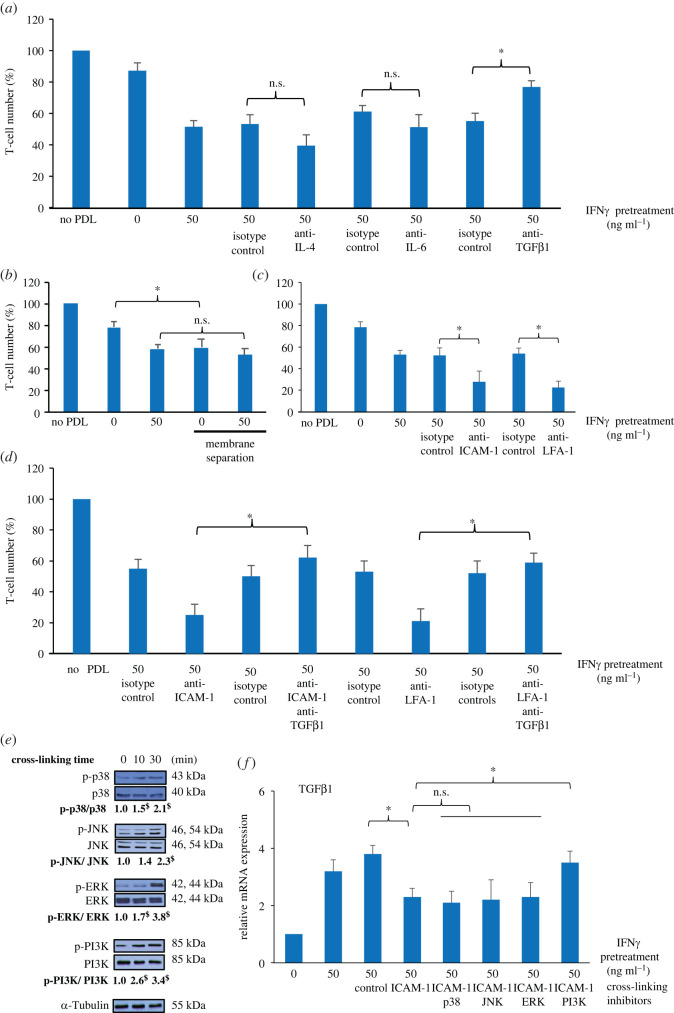
Effect of IFNγ-inducible mediators and ICAM-1-mediated direct cell contact on the suppression of T-cell viability by IFNγ-primed PDL cells. (*a–d*) Reduction of T-cell viability by IFNγ-inducible mediators (IL-4, IL-6 and TGFβ1), direct cell-to-cell contact and ICAM-1/LFA-1 were further investigated. PDL cells were pretreated with 50 ng ml^−1^ IFNγ for 48 h, and they were co-cultured with T cells at a PDL : T cell ratio of 1 : 5 for 24 h. Neutralizing antibodies against IL-4, IL-6, TGFβ1, ICAM-1 and LFA-1 were added to suppress the activity of these molecules while, in some experiments, transwell microporous membranes were used to prevent physical contact between these two cell types. Isotype control antibodies at the same concentrations as their corresponding specific antibodies were also used. The viability analysis of T cells was carried out using the trypan blue exclusion test, and the results are presented as the mean percentages ± s.d. of the number of viable T cells from three biological replicates, defined as 100% in the control group. (*e*) Western blot analysis of the expression of phosphorylated forms of p38, JNK, ERK and PI3 K, following cross-linking of ICAM-1 on IFNγ-primed PDL cells for the indicated times. The relative protein level of each of the phosphorylated forms, normalized by the respective total protein level, is shown as fold-change of the non-cross-linking sample (at 0 min), defined as 1.0. (*f*) The mRNA expression of TGFβ1 in IFNγ-primed PDL cells following cross-linking of ICAM-1 in the presence of chemical inhibitors specific to p38, JNK, ERK and PI3 K for 6 h. Data are presented as the mean fold-changes ± s.d. from three biological replicates, defined as 1.0 in the untreated control group. *p < 0.05. n.s., no significance. ^$^*p* < 0.05 versus non-cross-linking group.

Since IFNγ priming and the presence of direct cell contact appeared to differentially regulate T-cell viability and Th1/Th2 differentiation, the mechanisms involving IFNγ-inducible mediators and direct cell contact were therefore investigated. In figure 5*a*, neutralization of IL-4 and IL-6 only slightly enhanced IFNγ-primed PDL cell-mediated T-cell inhibition in co-cultures, whereas TGFβ1 blocking significantly rescued IFNγ-primed PDL cell-mediated T-cell suppression, resulting in an increased number of viable T cells from about 50% to the level about 80% of the T cell control. This suggests that the enhanced immunosuppression of IFNγ priming of PDL cells may be mediated via TGFβ1, but not IL-4 and IL-6. The results further showed that prevention of direct cell contact by a membrane separation significantly enhanced suppression of viable T cells by PDL cells but had little effect on that by IFNγ-primed PDL cells (figure 5*b*), suggesting that the enhanced immunosuppression of IFNγ-primed PDL cells was mainly mediated through soluble mediators, not the direct cell contact mechanism. In contrast to TGFβ1, blocking ICAM-1 and LFA-1 in co-cultures significantly increased the suppression of T-cell viability from about 50% to 20–25% of the control (figure 5*c*). When both neutralizing antibodies against ICAM-1/LFA-1 and TGFβ1 were added simultaneously in co-culture, the numbers of viable T cells in both conditions were about 60% of the T cell control, which were significantly higher than those with neutralization of only ICAM-1 or LFA-1 (approx. 20% of the T cell control) (figure 5*d*). This suggests an opposing effect between ICAM-1/LFA-1 and TGFβ1, but predominantly role of TGFβ1, on IFNγ-primed PDL cell-suppressed T-cell viability in co-cultures. We further tested whether activation of ICAM-1 on PDL cells affected the expression of TGFβ1. In figure 5*e*, intracellular signalling via p38, JNK1/2/3, ERK1/2 and PI3 K were activated by ICAM-1 cross-linking on IFNγ-primed PDL cells, as seen by increases in their phosphorylated forms following ICAM-1 ligation. The results also showed that while cross-linking of ICAM-1 significantly reduced TGFβ1 mRNA expression in IFNγ-primed PDL cells, an inhibitor specific to PI3 K, but not the three MAPKs, prevented ICAM-1 cross-linking-mediated inhibition of TGFβ1 expression in IFNγ-primed PDL cells (figure 5*f*). These data suggest that suppression of T-cell viability by IFNγ-primed PDL cells was prevented by ICAM-1-induced PI-3 K-mediated inhibition of TGFβ1 expression.

### 3.6. Induction of Th1 and Th2 differentiation by IFNγ-primed periodontal ligament cells was ICAM-1-dependent in IFNγ-primed periodontal ligament cells

**Figure 6.**
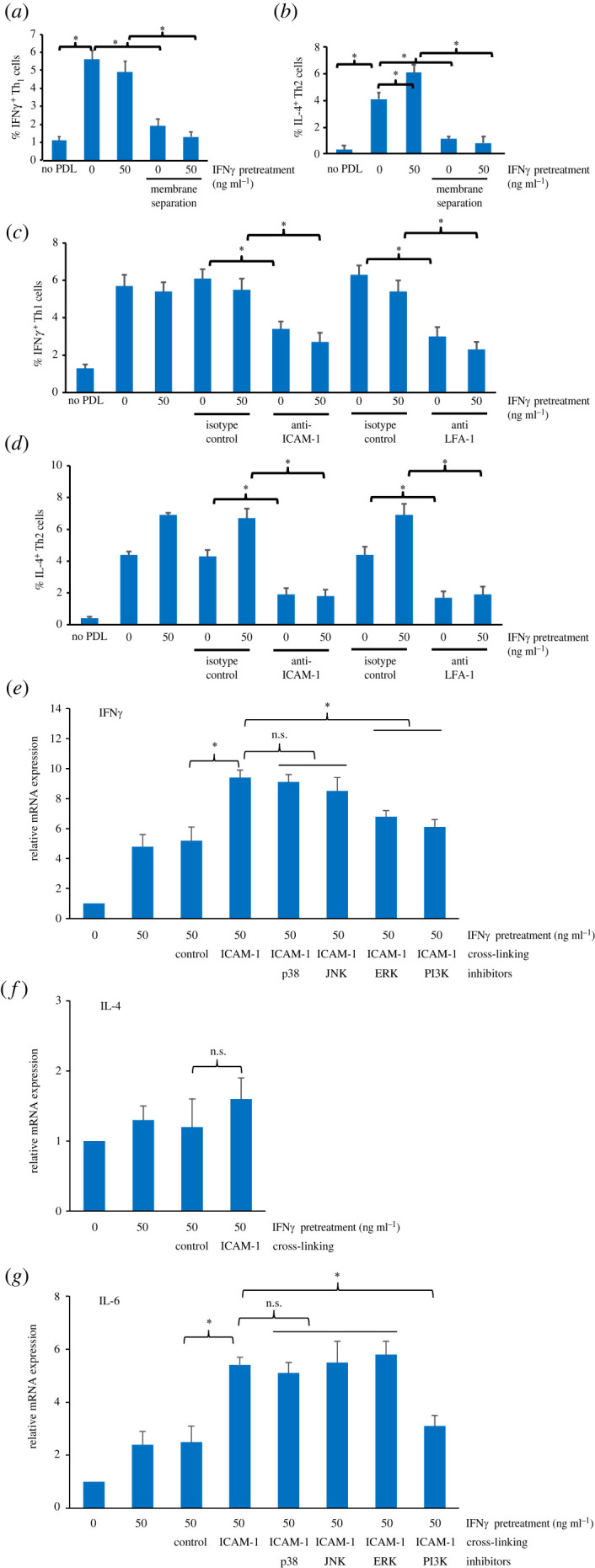
Effect of ICAM-1 signalling in IFNγ-primed PDL cells on Th1 and Th2 differentiation. PDL cells were pretreated with and without 50 ng ml^−1^ IFNγ for 48 h and co-cultured with T cells at a PDL : T cell ratio of 1 : 5 for 24 h with and without microporous membrane separation (*a,b*) or with and without blocking antibodies specific to ICAM-1 and LFA-1 (*c,d*) and the percentages of IFNγ^+^ Th1 cells and IL-4^+^ Th2 cells were determined by immunostaining and FCM. The results are presented as the mean percentages ± s.d. of three biological replicates. In (*e–g*), PDL cells were pretreated with 50 ng ml^−1^ IFNγ for 48 h, and they were subjected to cross-linking of ICAM-1 with and without chemical inhibitors specific to p38, JNK, ERK or PI3 K. After 6 h, the samples were subjected to RNA extraction and qPCR for the mRNA expression of IFNγ, IL-4 and IL-6. The mRNA expression is expressed as mean fold changes ± s.d. of three biological replicates, defined as 1.0 in the untreated control. *p < 0.05. n.s., no significance.

Since non-primed PDL cells and IFNγ priming enhanced Th1 and Th2 cell differentiation in co-cultures, precise mechanisms by which IFNγ-primed PDL cells influenced T-cell differentiation were examined. The results in figure 6 show that in the absence of direct physical contact between IFNγ-primed PDL-T CM, increased Th1 and Th2 cell subpopulations induced by IFNγ-primed PDL were significantly diminished (figure 6*a,b*, respectively). The results suggest a dominant role of direct cell-to-cell contact signalling in the induction of IFNγ^+^Th1 and IL-4 ^+^ Th2 differentiation by IFNγ-primed PDL. As with preventing physical cell contact, blocking ICAM-1 and LFA-1 in co-cultures also resulted in a marked reduction of IFNγ^+^ Th1 and IL-4 ^+^ Th2 subpopulations (figure 6*c,d*, respectively). Cross-linking of ICAM-1 on IFNγ-primed PDL cells stimulated the expression of ERK- and PI3 K-dependent IFNγ and PI3 K-dependent IL-6, but not IL-4, genes (figure 6*e–g*). IFNγ and IL-6 have been shown to stimulate the differentiation of T cells towards Th1 and Th2 cells, respectively [26–29].

### 3.7. IFNγ-inducible mediators derived from co-cultures of IFNγ-primed periodontal ligament cells and T cells inhibited macrophage activation and osteoclast formation

It has been shown that T lymphocytes can stimulate macrophage activation and osteoclast formation by their Th cell cytokines [30,31]. Since IFNγ-primed PDL cells decreased the number of viable T cells and the ratio of Th1/Th2 cells in co-cultures, cytokines released by IFNγ-primed PDL-T CM in co-cultures may suppress macrophage activation and osteoclast formation.

**Figure 7.**
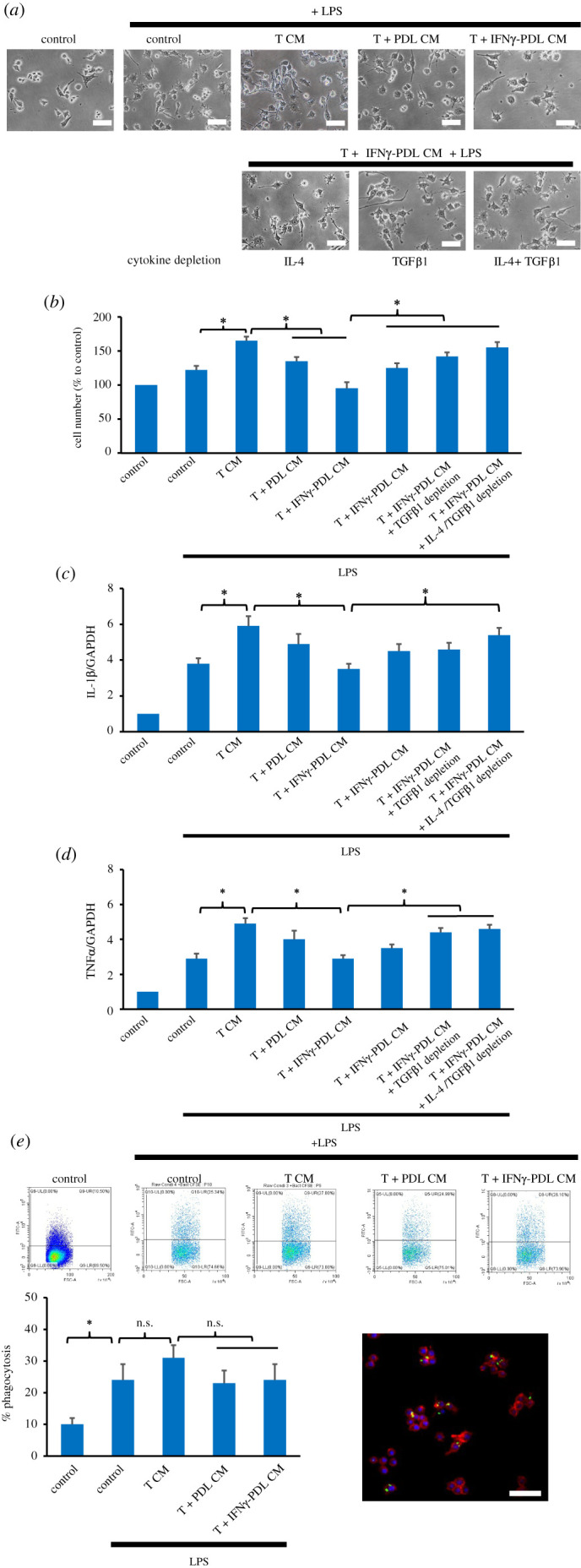
Effect of conditioned media from co-cultures of IFNγ-primed PDL cells and T cells on macrophage activation and phagocytosis. RAW 246.7 cells were treated with LPS (100 ng ml^−1^), and 1 : 1 diluted conditioned media collected from 24 h cultures of T CM, PDL-T CM) and IFNγ-PDL-T CM and after 24 h in culture, cell morphology, the number of viable cells and the expression of IL-1β and TNFα genes were determined by light microscopy (*a*), MTT assay (*b*) and qPCR (*c,d*), respectively. In (*e*), a phagocytosis assay, using heat-killed CFSE-stained *Streptococcus sanguinis* at a concentration of 10 bacteria per one RAW 246.7 cell for 60 min to allow phagocytosis, was carried out, and the phagocytosis level was determined by flow cytometry. Representative immunofluorescence is also shown to indicate cellular localization of CSFE-stained bacteria (green) in RAW 246.7 cells. DAPI-stained nuclei are shown in blue, and intracellular actin filaments are in red. The values are expressed as the mean percentages (fold changes) ± s.d. of three biological replicates, defined a 100% (1.0) in the control. *p < 0.05. n.s., no significance. Scale bar = 40 µm.

The functional importance of co-culture conditioned media on macrophage activation was determined, and the results showed that upon activation by LPS, RAW 264.7 macrophages changed their morphology and formed irregular shapes with accelerated spreading and pseudopodia, which was not affected by any of the conditioned media tested (figure 7*a*). However, the cell number seemed to be increased by T-cell-derived conditioned medium (T CM) and decreased by conditioned media from the co-cultures of non-primed PDL-T CM and of IFNγ-PDL-T CM. This was also supported by the results in figure 7*b*, which further demonstrates a significant reduction in the number of RAW 246.7 cells by PDL-T CM and IFNγ-PDL-T CM when compared with that in the T CM group. Depleting IL-4, TGFβ1 and both cytokines significantly reversed this inhibitory effect of IFNγ-PDL-T CM (figure 7*b*). While T CM upregulated the mRNA expression of IL-1β and TNFα, IFNγ-PDL-T CM inhibited this T CM effect at least partly by the presence of IL-4 and TGFβ1 (figure 7*c,d*). In figure 7*e*, FCM results showed that 10% of untreated RAW 246.7 cells phagocytosed heat-killed CFSE-stained bacteria, and 26% of LPS-induced RAW 246.7 cells possessed the phagocytic ability. All the conditioned media tested did not significantly affect the phagocytosis ability of LPS-induced RAW 246.7 cells. The representative confocal fluorescence micrograph in figure 7*e* demonstrates phagocytosed CFSE-stained bacteria (green dots) localized in LPS-induced RAW 246.7 cells. The results suggest that IFNγ-PDL-T CM reduced cell number, the expression of IL-1β and TNFα, but not phagocytosis, of LPS-induced RAW 246.7 cells via IL-4- and TGFβ1-dependent mechanisms.

**Figure 8.**
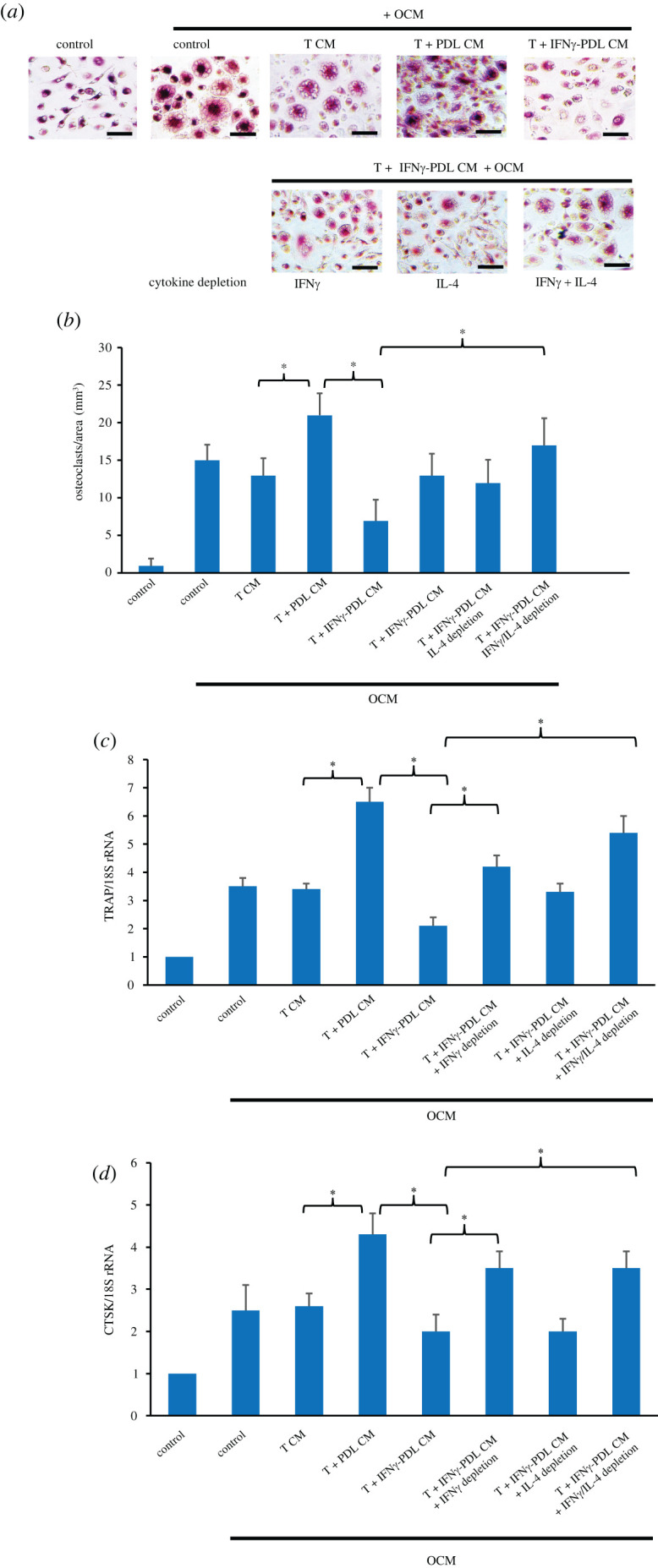
Effect of conditioned media from co-cultures of IFNγ-primed PDL cells and T cells on osteoclast formation. PBMCs were cultured with and without an osteoclastic medium (OCM), consisting of 25 ug ml^−1^ MCSF and 25 ug ml^−1^ RANKL, and 1 : 1 diluted conditioned media collected from 24 h cultures of T cells alone (T CM), PDL cells and T cells (PDL-T CM) and IFNγ-primed PDL cells and T cells (IFNγ-PDL-T CM) and after 14 days in culture, the presence of TRAP^+^ multinucleated cells, the number of TRAP^+^ multinucleated cells and the expression of TRAP and CTSK genes were determined by TRAP staining followed by light microscopy (*a*) and analysis by counting osteoclasts per area (mm^3^) (*b*) and qPCR (*c,d*), respectively. The values are expressed as the mean fold-changes ± s.d. of three biological replicates, defined as 1.0 in the control. *p < 0.05. Scale bar = 40 µm.

Regulation of osteoclast formation by the conditioned media was evaluated using PBMCs, which under stimulation with OCM, formed large TRAP^+^ osteoclasts *in vitro*. In contrast to the effect on macrophage activation, T CM had little effect on the formation of osteoclasts, and PDL-T CM significantly increased the number of osteoclasts (figure 8*a*). IFNγ-PDL-T CM resulted in marked suppression of osteoclast formation, which was reversed by depletion of IFNγ and IL-4 in the conditioned medium. However, only the reversal effect of simultaneous depletion of both cytokines appeared to be statistically significant (figure 8*b*). The role of IFNγ and IL-4 present in IFNγ-PDL-T CM in osteoclast inhibition was further supported by the results in figure 8*c,d*, which showed that IFNγ-depleted and IFNγ/IL-4-depleted IFNγ-PDL-T CM significantly prevented IFNγ-PDL-T CM-reduced osteoclast formation. The results suggest that IFNγ-PDL-T CM significantly inhibited osteoclast formation, at least partly by its presence of IFNγ and, to a lesser extent, IL-4.

## 4. Discussion

PDL cells play an important role in tissue homeostasis [1], but the precise mechanisms by which PDL cell-mediated tissue homeostasis during tissue inflammation are not yet fully understood. The present results have shown that IFNγ-primed PDL cells possessed enhanced immunosuppression by suppressing T lymphocytes and directing T-lymphocyte differentiation towards a lower Th1/Th2 cell ratio. Suppression of T-cell viability by IFNγ-primed PDL cells appeared to be mainly mediated by IFNγ-inducible secreted mediators, which was prevented in the presence of direct cell contact, probably by ICAM-1-induced PI3 K-mediated TGFβ1 expression in PDL cells. By contrast, ICAM-1 activation augmented IFNγ priming-induced IFNγ and IL-6 expression in PDL cells (via ERK and PI3 K) and mediated the effect of IFNγ-primed PDL cells on Th1/Th2 cell differentiation. Compared with non-primed PDL cells, the interaction between IFNγ-primed PDL cells and T lymphocytes favoured IL-4/TGFβ1-dependent suppression of macrophage activation and IFNγ/IL-4-dependent osteoclast inhibition. The present findings provide an important insight into the mechanisms by which PDL cells and IFNγ may regulate PDL tissue homeostasis in the inflammatory microenvironment and thus facilitate tissue healing following injury. These findings also lay the groundwork for future *in vivo* studies investigating the role of TGFβ1 and ICAM-1 in periodontal tissue regeneration.

MSCs, including those present in the PDL tissue, possess immunosuppressive activity. It is, however, possible that non-MSC fibroblasts may have the potential to inhibit immune cell responses, as previously been reported for dermal and gingival fibroblasts [32,33]. PDL cells, used in the present study, are heterogeneous, containing both an MSC population and non-MSC populations (such as fibroblasts and progenitors of cells found in periodontal tissues), supporting the immunomodulatory role of PDL cells, as also shown in the present study. While previous studies in PDL-derived MSCs demonstrate that the immunosuppression is mediated mainly via soluble mediators they secrete [12], the present results suggest that the interaction between IFNγ-primed PDL-T CM may also play an important role in the presence of secreted soluble mediators necessary for immunosuppression, as denoted by the lack of suppressive effect of non-primed PDL cells and IFNγ-primed PDL cells on T-cell viability (figure 2*a*). This emphasizes that in PDL cells, enhanced immunosuppression requires activation, which may occur via cell-cell interaction or via the secreted molecules.

The viability and proliferation of T cells can be regulated by TGFβ [34]. The present study has provided evidence that increased PDL cells-derived TGFβ1 production by IFNγ priming enhanced the anti-proliferative effect of PDL cells on T cells. In PDL cells, although IFNγ primming stimulated the expression of IFNγ, IL-6 and TGFβ1 genes and their secreted protein levels, only TGFβ1 seemed to play a role in suppressing the number of viable T cells. This is supported by a previous study showing that only certain inflammatory cytokines influence T- cell responses, with TGFβ being most dominant, followed by IL-6 and IL-4 [35]. In addition, the role of PI3 K-dependent ICAM-1-downregulating TGFβ1 gene expression in reversing the inhibitory effect of IFNγ-primed PDL cells was shown in the present study, for our knowledge for the first time. This suggests that PI3 K-mediated TGFβ1 gene expression in IFNγ-primed PDL cells may serve as a promising therapeutic target for controlling inflammatory conditions, especially in the PDL tissue. Moreover, the inflammatory and T-cell phenotypes observed in T-cell-specific TGFβ1—deficient mice are less severe than those of mice completely lacking TGFβ1 [36,37], suggesting that TGFβ1 produced by cell types other than T cells, such as by PDL cells—a mesenchymal cell type present in the oral cavity, may also be involved in the regulation of T cells. These hypotheses warrant further studies.

ICAM-1 expression in antigen-presenting cells is essential for efficient T-cell activation [38,39]. Moreover, ICAM-1 clustering is required for LFA-1-mediated T-cell activation [40,41]. An important role of PDL ICAM-1 cross-linking in the viability of T cells was demonstrated in the present study, to our knowledge for the first time. In IFNγ-primed PDL cells, ICAM-1-induced PI3 K-mediated inhibition of TGFβ1 expression rescued T-cell viability against an inhibitory effect of IFNγ-primed PDL cells, providing further insight into the mechanism of ICAM-1-mediated T-cell activation indirectly by inhibiting T-cell suppression. This also suggests a subsidiary role of the cell-to-cell contact mechanism in T-cell inhibition compared with that of secreted biological mediators.

Multiple signalling pathways and sophisticated crosstalks tightly control T-cell differentiation. The present results showed that IFNγ-primed PDL cells stimulated T-cell differentiation towards Th1 and Th2 lineages at least in part through an ICAM-1-dependent mechanism, possibly via up-regulation of IFNγ and IL-6 in IFNγ-primed PDL cells. In addition to its role in T- cell viability, the role of PDL ICAM-1 signalling in T-cell differentiation was demonstrated, to our knowledge for the first time, in the present study. IFNγ-primed PDL cells induced T-cell differentiation towards Th1 and Th2 lineages via an ICAM-1-dependent mechanism. Previous *in vitro* and *in vivo* studies report the variable immunoregulatory effect of MSCs on Th1/Th2 cell differentiation [42–48]. The reason for this discrepancy remains unclear. The time of co-culture, the level of T-cell activation, the local microenvironment, and disease status have also been proposed to influence the immunomodulatory effect of MSCs on T-cell differentiation both *in vitro* and *in vivo* models [42,49]. It is also possible that cells within PDL tissue may have frequently been exposed to oral microorganisms and thus being well trained to activate the protective response by stimulating Th1 cell differentiation, as previously been suggested for MSCs derived from other tissues, which can gain their effects to protect the tissue from injury in different conditions [17,49–51]. However, the ratio of Th2 cells to Th1 cells in the present study significantly increased when co-cultured with IFNγ-primed PDL cells, again suggesting a possible role of IFNγ-primed PDL cells in suppressing further inflammatory reaction (Th1 response) and driving towards regeneration (Th2 response). Further studies are undoubtedly required to investigate this.

MAPKs ERK, p38 and JNK have previously been shown to mediate several functions of ICAM-1, and all three MAPKs were activated following ICAM-1 stimulation, either via leukocyte attachment or antibody-mediated clustering [15,16]. JNK activity is critical for ICAM-1-mediated F-actin rearrangements [15], which is one of the key events in T-cell activation and differentiation [52]. Although all three MAPKs and PI3 K pathways were activated after ICAM-1 activation in PDL cells used in the present study, only ERK and PI3 K appeared to be involved in the upregulation of IFNγ and IL-6. These cytokines have been shown to stimulate the differentiation of T cells towards Th1 and Th2 cells, respectively [26–29,53,54]. Whether the increased expression of IFNγ and IL-6 genes in PDL cells play a role in ICAM-1 mediated Th1 and Th2 differentiation warrants further studies.

PDL-derived MSCs obtained from an inflamed PDL tissue regulate PDL tissue homeostasis during inflammation by suppressing the activity of peripheral blood mononuclear cells (PBMNCs) [55]. Compared with cells isolated from a healthy PDL tissue, these cells possess diminished inhibitory effects on PBMNC proliferation and the stimulation of regulatory T-cell and IL-10 production [56]. The present findings strongly support the role of IFNγ in enhancing immunosuppression by PDL cells. These inflamed PDL-derived MSCs preserve their proliferative ability, multipotency and regenerative potential, including its osteogenic potency, and the cells undergoing osteoblast differentiation also maintain their suppressive function on T-cell proliferation [57,58]. For proper T-cell proliferation and differentiation into certain lineages to occur, it has been shown that cytokines produced in the environment must be present at certain levels and proportions, with TGFβ, IL-6 and IL-4 being the most dominant mediators [35]. This also supports the importance of PDL cell-derived IFNγ-induced soluble mediators in regulating T-cell proliferation and differentiation. Together with our findings, such information raises a possibility that among inflammatory cytokines present in the inflamed microenvironment, IFNγ may play a role in a transition from an inflammation phase to a regeneration phase during PDL tissue healing after injury.

Several lines of evidence suggest an important role of LFA-1/ICAM-1 engagement in inducing T-cell activation and differentiation. Dysregulation of the expression of ICAM-1 ligand LFA-1 in T cells can lead to changes in T-cell activation and differentiation [59,60]. LFA-1/ICAM-1 cross-linking has been shown to induce human IFNγ^+^Th1 cell differentiation and functions [61]. In the present study, the results reveal two new and important roles of ICAM-1 signalling in IFNγ-primed PDL cells. First, the indirect role of ICAM-1 in the viability of T cells is evident by its effect against the anti-proliferation of T cells by IFNγ-primed PDL cells. This is at least in part mediated by downregulation of TGFβ1 gene expression in IFNγ-primed PDL cells. Second, ICAM-1 induced T-cell differentiation towards Th1 and Th2 lineages via upregulation of IFNγ and IL-6 in IFNγ-primed PDL cells.

The biological importance of IFNγ-inducible mediators derived from co-cultures of IFNγ-primed PDL-T CM was demonstrated by their ability to inhibit LPS-induced M1 macrophage activation and osteoclast formation. Cytokines derived from Th cells have been involved in macrophage activation and osteoclast formation [30,31,62]. Prolonged inflammation and subsequent bone resorption may cause imbalance homeostasis of PDL and bone tissues. Activation of macrophages is dependent on at least two main pathways, including that initiated by IFNγ and those sensitizing the macrophage to respond to IFNγ [62]. Thus, IFNγ secreting Th1 cells directly activate M1 macrophages, and IL-10 secreting Th2 cells can deactivate M1 macrophages by promoting M2 polarization. In the present study, IFNγ-primed PDL cells appeared to increase the proportion of Th2 to Th1, thereby suppressing M1 macrophage activation via increased levels of IL-4 and TGFβ1. It is possible that IL-4 and TGFβ1 present in the culture promoted M2 macrophage polarization, as previously reported [63,64], thus decreasing the M1 macrophage number and the gene expression of cytokines IL1-*β*1 and TNFα. It is noteworthy that IFNγ-primed PDL cells did not change the macrophage phagocytic activity, possibly maintaining the protective immunity against microbial infection. This warrants further *in vivo* studies to elucidate the involvement of cells of monocyte/macrophage lineages in homeostasis of PDL and bone tissues following injury or infection.

Following injury, the formation of osteoclast induced by prolonged inflammation causes bone destruction and reduced proper bone remodeling. The presence of both IFNγ and IL-4 in the co-culture between T cells and IFNγ-primed PDL cells were necessary for the inhibitory role in the suppression of osteoclast formation. Such function of these cytokines was supported by previous studies [9,10,65]. Moreover, in the absence of infection, IFN-γ represents its primary effect as an anti-osteoclastic activity under an inflamed microenvironment [66]. It is, however, noteworthy that our unpublished data (W. Singhatanadgit, S. Kitpakornsanti 2022) showed that T cells did not significantly affect the expression of genes associated with mineralization, i.e. a proteolytic enzyme alkaline phosphatase and an extracellular matrix protein type I collagen by non-primed PDL and IFNγ-primed PDL cells. This suggests an important role of PDL cells and IFNγ-inducible mediators in regulating bone homeostasis of the inflammatory PDL tissue.

## 5. Conclusion

In conclusion, the results have shown, for the first time to our knowledge, that PDL cells and IFNγ priming differentially control T-cell viability and differentiation via IFNγ-inducible mediators and ICAM-1-mediated direct cell contact, suggesting their central role in PDL homeostasis, favouring a shift of an inflammatory/destructive condition towards a regenerative healing condition. The study proposes that certain IFNγ-inducible mediators, specifically TGFβ1, and ICAM-1 are promising therapeutic targets for enhancing periodontal tissue regeneration.

## Data Availability

Data are available from the Dryad digital repository at https://doi.org/10.5061/dryad.qz612jmh5 [67]. Data is also available in the electronic supplementary material [68].
